# Rational design of HIV vaccines and microbicides: report of the EUROPRISE annual conference 2011

**DOI:** 10.1186/1479-5876-10-144

**Published:** 2012-07-11

**Authors:** Nicolas Ruffin, Marie Borggren, Zelda Euler, Fabio Fiorino, Katrijn Grupping, David Hallengärd, Aneele Javed, Kevin Mendonca, Charlotte Pollard, David Reinhart, Elisa Saba, Enas Sheik-Khalil, Annette Sköld, Serena Ziglio, Gabriella Scarlatti, Frances Gotch, Britta Wahren, Robin J Shattock

**Affiliations:** 1 Department of Microbiology, Tumor and Cell Biology, Karolinska Institutet, Nobels väg 16, Stockholm, 171 77, Sweden; 2 Department of Laboratory Medicine, Lund University, Sölvegatan, Lund, 223 62, Sweden; 3 Department of Experimental Immunology, Landsteiner Laboratory of Sanquin and the Academic Medical Center, University of Amsterdam, Meibergdreef, Amsterdam, 1105 AZ, The Netherlands; 4 Department of Biotechnology, University of Siena, Viale Bracci, Siena, 53100, Italy; 5 Institute of Tropical Medicine, Nationalestraat 155, Antwerp, 2000, Belgium; 6 Leibniz Institute for Primate Research, German Primate Centre, Kellnerweg, Goettingen, 37077, Germany; 7 Polymun Scientific GmbH, Donaustraße 99, 3400, Klosterneuburg, Austria; 8 Department of Biotechnology, University of Natural Resources and Life Sciences, Muthgasse 11, 1190, Vienna, Austria; 9 AIDS Immunopathogenesis Unit, Department of Immunology, Transplantation and Infectious Diseases, San Raffaele Scientific Institute, via Olgettina, Milan, 20132, Italy; 10 Center for Infectious Medicine, Karolinska Institutet, Karolinska University Hospital, Huddinge F59, Stockholm, 141 86, Sweden; 11 Department of Life and Reproduction Sciences, University of Verona, Strada Le Grazie, Verona, 37134, Italy; 12 Viral Evolution and Transmission Unit, Department of Immunology, Transplantation and Infectious Diseases, San Raffaele Scientific Institute, Via Olgettina, Milan, 20132, Italy; 13 Department of Immunology, Imperial College London, Fulham Road, London, SW10 9NH, UK; 14 Centre for Infection, Department of Clinical Sciences, St George's, University of London, Cranmer Terrace, London, SW17 0RE, UK

**Keywords:** HIV, Vaccine, Microbicide, PrEP

## Abstract

Europrise is a Network of Excellence supported by the European Commission within the 6th Framework programme from 2007 to 2012. The Network has involved over 50 institutions from 13 European countries together with 3 industrial partners and 6 African countries. The Network encompasses an integrated program of research, training, dissemination and advocacy within the field of HIV vaccines and microbicides. A central and timely theme of the Network is the development of the unique concept of co-usage of vaccines and microbicides. Training of PhD students has been a major task, and some of these post-graduate students have here summarized novel ideas emanating from presentations at the last annual Europrise meeting in Prague. The latest data and ideas concerning HIV vaccine and microbicide studies are included in this review; these studies are so recent that the majority have yet to be published. Data were presented and discussed concerning novel immunisation strategies; microbicides and PrEP (alone and in combination with vaccines); mucosal transmission of HIV/SIV; mucosal vaccination; novel adjuvants; neutralizing antibodies; innate immune responses; HIV/SIV pathogenesis and disease progression; new methods and reagents. These – necessarily overlapping topics - are comprehensively summarised by the Europrise students in the context of other recent exciting data.

## Introduction

The 2011 Network Annual Conference of Europrise was held from 14th to 17th November in Prague, the Czech Republic, and was attended by 100 partners from 31 European institutions, two members of the advisory board, ten affiliated partners and eight invited guests. Scientific data from earlier meetings were summarized previously [1,2]. New important data from oral and poster presentations were discussed by all collaborators at the conference and are summarised here by our PhD students. All participants at the meeting were greatly encouraged and excited by positive data from recent microbicide and vaccine trials, in the context of which the meeting’s eight sessions dealt with the latest European clinical trials of pre-exposure prophylaxis (PrEP) with vaccines, microbicides and drugs in both man and animal models, using conventional and novel strategies, and aimed at inducing measureable innate and specific immune responses, both cellular and humoral.

One of the outstanding features of the Europrise Network of Excellence has been a work package dedicated to a PhD student training programme. In this context, young researchers have made exchange visits between laboratories of the Network and have attended dedicated workshops and conferences. During Europrise’s lifetime, 35/64 students from 30 laboratories have completed their postgraduate studies, 20 of these students were directly funded by the Europrise programme.

## HIV vaccines

Several strategies to hasten the development of an effective HIV vaccine were presented. These strategies included the production of novel vector platforms, the identification of potent adjuvants, the development of delivery devices and novel challenge models. A description of these novel immunization strategies is given, and their implications are discussed.

### Clinical trials

#### Immunological correlates of protection

The RV144 Phase III trial is the only trial in which the clinical efficacy, estimated at 31%, of an HIV vaccine has been demonstrated in man [3]. This prime-boost strategy was an attempt to combine an avipox recombinant vector expressing human immunodeficiency virus type 1 (HIV-1) Gag, Pro, with a bivalent gp120 protein boost (AIDSVAX B/E) [3]. This trial involved 16,400 Thai subjects. The modest efficacy signal in the RV144 trial has enabled a formal search for correlates of risk (increased or decreased risk of infection within the vaccinated cohort), involving a pre-specified plan of six primary and a large number of exploratory analyses. Analyses compare immune responses in vaccinated infected persons with those in vaccinated, uninfected persons [4,5]. Jerome Kim from the Walter Reed Army Institute of Research presented data showing that 2 of the 6 pre-specified primary analyses, corrected for multiple comparisons, were identified as correlates of risk that may be used as hypotheses for future testing. The first was a measure of the magnitude and breadth of anti-HIV Env IgA binding that was directly correlated with infection (RR 1.54; p = 0.027), although no enhancement of infection was seen compared to the placebo groups. The second was an indirect correlation between IgG binding to the V1V2 loop of gp120 and a 43% reduction in the HIV infection rate (RR 0.57 p = 0.015). Sieve analyses of breakthrough viruses, as well as antibodies from vaccinated uninfected volunteers, suggest that antibodies against the V2 loop may be associated with escape mutations in breakthrough viruses. Taken together, these findings support the original modest efficacy of the RV144 trial, and suggest hypotheses that can be tested in future animal challenge experiments [4].

Scientists from IDIBAPS (Institut d´Investigacions Biomèdiques August Pi in Sunyer and University of Barcelona) reported the results of the phase I clinical trial RisVac02 [6]. The study’s purpose was to evaluate the safety and immunogenicity of a modified vaccinia virus Ankara (MVA) vector expressing monomeric gp120 and the fused Gag-Pol-Nef polyprotein of clade B (MVA-B). Thirty HIV-1 negative volunteers were given three intramuscular injections of MVA-B or placebo at weeks 0, 4 and 16 and followed for one year. The vaccine was safe and well tolerated: a number of adverse events were reported but they either were not related to the vaccination or were injection site reactions. The majority of the individuals (75 %) exhibited broad and polyfunctional CD8+ and CD4+ T-cell responses, especially directed against Env. Responses were durable, being detectable during the entire follow-up period, and were mainly found within the effector memory T-cell subset. Antibody responses to Env were induced in almost all the vaccinees and HIV neutralizing activity against BX08, tier 1, was observed in 33% of the volunteers [7]. Taken together, these findings suggest MVA-B as a promising HIV-1 vaccine candidate. Further studies are needed to assess the boosting effects of recombinant proteins. These results reflect the wider recognition that pox-based vectors represent promising components of potential HIV vaccines, the basis for the formation of the Pox Protein Public Private Partnership (P5, by Sanofi and Novartis, see AVAC.org) initiated with the objective of building on these and other important results to develop further pox-protein vaccines [8].

Another Europrise study (HIVIS) was reported which has advanced prophylactic vaccination by priming with multigene DNA, followed by MVA-CMDR of the type CRF A_E; thereafter the intention is to boost with the Env glycoprotein subtype C, the novel concept of prime-boost-boost. Consecutive development of cellular and then antibody responses to many HIV-1 subtypes was described [1,2,9]. A serological study to determine the breadth and magnitude of antibody responses to inserts in DNA, viral vector or protein components of the HIVIS vaccine is underway, supported by an innovation grant from Europrise.

Susan Barnett from Novartis described the planned development within the context of the Pox Protein Public Private Partnership (P5) of a proof-of-concept trial in South Africa, building on the findings of the RV144 trial but using clade C inserts and protein (gp120). The aim is to develop new envelope proteins and reagents for the subtype C HIV-1 epidemic. A pox virus (ALVAC) prime developed by Sanofi-Pasteur will contain the highly immunogenic ZM96 gp120–TM [10] and the boost will contain two subtype C gp120 proteins (TV-1 and 1086 C) formulated with the licensed potent vaccine adjuvant MF59. Use of gp120 monomers instead of trimers has been prioritised in order to make comparisons with data from the RV144 trial [3].

Data were presented on a novel T-cell immunogen, termed HIVconsv, designed by Tomas Hanke and Andrew McMichael (University of Oxford) to address HIV-1 diversity. Fourteen of the most conserved regions of Gag, Pol, Vif and Env have been assembled into one synthetic DNA construct. To ensure coverage of the virus diversity, each consensus fragment was derived from different HIV-1 clades [11]. The sequence coding for the HIVconsv immunogen has been inserted into plasmid DNA, MVA and into non-replicating adenovirus of chimpanzee origin ChAdV-63 [12]. The three candidate vaccines administered in combination were highly immunogenic in mice and macaques. A phase I/IIA clinical trial (HIV-CORE002) is currently underway to evaluate their safety and immunogenicity in healthy adults. Preliminary results suggest that the HIVconsv-based vaccines are safe and able to induce T-cell responses.

### Studies of attenuated viruses and virus-like particles

In order to be useful as immunogens, inactivated virions should preserve their immunogenicity and be safe for the patient. Plana and collaborators (Hospital Clinic-IDIBAPS, Barcelona) investigated the immunogenicity of a reverse transcriptase (RT) defective virion (NL4-3/ΔRT). Using stored lymphocytes from asymptomatic HIV-positive patients, they showed that NL4-3/ΔRT viruses induced T-cell responses in vitro in 56% of the individuals tested, while only 24% of them were responsive to the NL4-3 wild type virions inactivated with aldrithiol-2 [13]. The immune responses were significantly stronger against the RT-defective viral particles and included both CD4+ and CD8+ restricted responses. The results encourage further exploration of NL4-3/ΔRT viruses as additional reagents for screening HIV-1 specific responses in HIV seropositive individuals and vaccinees.

Further interrogation of the mechanisms of protection provided by attenuated viruses was reported in non-human primate (NHP) studies. Martin Cranage presented work performed by Maria Manoussaka as part of a collaboration with Neil Almond’s team at NIBSC and Ben Berkhout’s team at AMC, demonstrating that infection of macaques with SIVmac239Δnef or a doxycycline (dox)-dependent conditional replication variant of SIVmac239Δnef, designated SIVrtTA, increases the proportion of circulating T effector memory (Tem) (CD28-CD95 + CCR7-) cells with a concomitant reduction of T central memory (Tcm) (CD28 + CD95 + CCR7+) CD4+ and CD8+ cells only under replication-permissive conditions. Similar changes following infection were found in gut mucosal-homing (α4 + β7+ and β7+) T-cells but the changes were also maintained after doxycycline withdrawal. Also, global phenotype data of polyfunctionality of SIV-specific T-cells in the periphery and in the gut of the animals were presented. These data may contribute to our understanding of the mechanisms of resistance to superinfection.

The mechanisms behind protection induced by live attenuated SIV are still unclear. One of the theories is that the failure to superinfect ‘immunized’ monkeys is due to competition for available target cells rather than immune control. To uncouple replication from immune response, Oliver Hohn (Robert Koch Institute) produced a 181 bp nef-deletion version of Klaus Überla’s SIVmac RT-SHIV that expresses the HIV reverse transcriptase. Unlike wild-type SIVmac, this virus was highly susceptible to the antiviral compound nevirapine. Eight rhesus macaques were infected with nef RT-SHIV; after allowing the immune response to develop for 40 weeks, four were given a daily dose of nevirapine (together with four 'unvaccinated' control animals). At 48 weeks, all 16 monkeys (vaccinated and unvaccinated, with and without nevirapine treatment) were challenged with wild-type SIVmac239. During the vaccination phase, all animals developed virus specific B- and T-cells. Peak viral load in vaccinated macaques after challenge was 2-3 orders lower than in control animals, and by week 10 all vaccinated animals were virus negative. Most importantly, there was no evidence that suppressing virus replication by nevirapine diminished the ability of the immunized macaques to resist infection by the challenge virus. These exciting results led to the conclusion that the suppression of virus replication in vivo was not the result of competition for infectable targeT-cells through active replication of the vaccine virus.

An alternative approach to attenuated viruses is the development of virus-like particles (VLP). This proved to be successful for the group of Buonaguro et al (Natl. Cancer Institute Pascale, Naples) which used the insect baculovirus system to produce VLP. These VLP vaccines, which are non-infectious and safe, show promise with or without HIV DNA priming [14].

### Preclinical challenge models

Anti-viral CD8+ T-cell responses are commonly studied mechanisms, thought to play a protective role during HIV-1 disease progression. Several presentations at the Europrise meeting focused on novel T-cell vaccines in NHP models, with a low-dose repeated rectal challenge. Such models may give us better insight into the potential clinical utility of vaccines following a physiologically relevant challenge.

Neil Almond from NIBSC presented data on a novel vaccine designed to promote potent T-cell responses to multiple epitopes, based on a strategy designed and developed by Steve Patterson (Imperial College). Mauritian cynomolgus macaques were immunized intra-dermally with recombinant DNA and boosted with a recombinant non-replicating Adenovirus expressing wild type SIV full length Gag, with an ubiquitin sequence fused to the amino terminus, or a fragmented SIV Gag gene with an ubiquitin sequence fused to the amino terminus of each fragment. Animals were challenged repeatedly intra-rectally with low-dose SIVmac251. No difference in protection was seen between the three groups of vaccinated animals receiving the fragmented or full length SIV Gag. However, there was evidence that vaccinated macaques were less likely to acquire infection compared with naive challenge controls, though the effect did not quite achieve significance at the 5% level. These data suggest that modification of SIV Gag based antigens by either ubiquitin fusion strategies or fragmentation provide no significant advantage to to a vaccine that provided mildly beneficial effect. Additional studies are underway to compare the immunogenicity of these different vaccines as well as alternative routes of vaccination.

In a further study, reported by Christiane Stahl-Hennig from the German Primate Center, rhesus macaques were subcutaneously primed with single-cycle immunodeficiency virus (SCIV), followed by either an oral spray application or an intramuscular injection of either a recombinant Adenovirus (rAd) expressing SIV Gag/Pol and Env, or a recombinant fowlpox virus (rFPV) expressing SIV Gag and Env. Animals were then repeatedly challenged intrarectally with low-dose SIVmac251. The vaccination regimen with immunization with oral rFPV followed by the systemic rAd was superior to the reverse strategy, as shown by the delayed acquisition of highly pathogenic SIV and reduced viral load post infection.

Klaus Uberla (University of Bochum) presented a strategy designed to direct antigens to dendritic cells (DC) and enhance T-cell antigen presentation, using a novel modality where Gag p27 is fused with a single-chain antibody that is specific for the DC-restricted antigen uptake receptor, DEC205. Macaques received the vaccine intramuscularly and were later boosted with virus-like particles to induce a humoral immune response. Somewhat counter-intuitive results showed that animals vaccinated with control antibodies coupled to Gag p27 made stronger IFNγ CD4 and CD8 T-cell responses than animals vaccinated with p27 fused to the DEC205 antibodies. After repeated low-dose rectal challenges there was a trend to faster infection in the group with the higher IFNγ responses and animals with a high IFNγ response were infected significantly faster than poor responders. These findings raise questions as to whether vaccine-induced immune responses may under some circumstances lead to increased risk of acquiring SIV infection.

The role of APOBEC 3 G (apolipoprotein B mRNA editing cytokine deaminase) and AID (activation induced deaminase), innately expressed by B-cells, in protection against SHIV infection was discussed by Thomas Lehner (GKT, London). Macaques were immunized with recombinant MHC constructs linked to HIV/SIV antigens, HSP70 and dextran. Results showed significant upregulation of AID in B-cells and upregulation of APOBEC 3G in CD27+ memory B-cells and CD4+ effector memory T-cells in immunized animals. Following challenge with SHIV SF162.P4 viral load was inversely correlated with AID in B-cells and APOBEC 3 G in B- and T-cells of infected animals, suggesting that the MHC/SHIV vaccine construct stimulated parallel responses by the two deaminases that might be involved in pre-entry and post-entry control of SHIV replication [15].

In a SHIV-based model, presented by Mark Page (NIBSC), macaques were vaccinated with an HIV-1 W61D rgp120 envelope construct and challenged intravenously with heterologous viruses. Neutralizing antibodies were detected against rgp120 homologous to the vaccine, but only limited neutralization was seen against the two heterologous SHIV challenge strains. Protection against infection correlated with T-cell proliferative responses specific for peptides present in HIV-1 W61D rgp120. However, transfer of serum from the vaccinated animals with measureable titres of neutralizing antibodies to naive animals did not confer protection, highlighting a putative important role of CD4+ T-cells in protection from HIV-1 infection.

Measles vaccine vectors have been reported as a novel strategy with a potential to deliver heterologous antigens into the antigen-processing pathways, promoting strong CD4+ and CD8 + T-cell responses [16]. In this context, Richard Stebbings (NIBSC) reported an immunogenicity study to characterize cellular immune responses induced by a recombinant measles vector carrying inserts for HIV-1 Clade C Gag, RT and Nef (MV1-F4) in cynomolgus macaques. A significant increase in the T-cell responses to the HIV insert was detected in animals immunized with both low and high dose MV1-F4. Of interest, the addition of a second boost further increased CD4+ T-cell responses in both groups despite the presence of high titres of measles neutralising antibodies, although this was not the case for CD8+ T-cell responses.

## Novel immunization strategies

A major hurdle in the development of HIV-1 vaccines is the construction of immunogens capable of inducing antibodies that can broadly neutralize HIV-1. In order to prevent initial infection of host cells or limit early events of viral dissemination, neutralizing antibodies must target the surface envelope glycoproteins of many human immunodeficiency viruses, which are highly variable in sequence and structure [17]. Indeed, as principal viral target of neutralizing antibodies, the HIV-1 viral spike has evolved to evade antibody-mediated neutralization. Anna-Lena Spetz (Karolinska Institutet) presented data on a new vaccination strategy under development to overcome the high diversity of HIV-1 by inducing immune responses towards relatively conserved regions of gp120 and gp41. Since the variable epitopes of Env are generally more immunogenic than the conserved parts, immune responses are preferentially induced to variable regions. In order to focus B-cell responses to conserved neutralizing epitopes of Env, Spetz et al. applied a heterologous prime-boost strategy consisting of a DNA prime comprising a mix of three HIV-1 Env clade B gp140 constructs, followed by a boosting with a very different Env trimeric gp140 protein, administered to rabbits. They hypothesized that by mixing three different envelopes during the DNA prime, the concentration of antigen derived from the conserved regions would be higher than that from the more variable regions. Boosting with a different Env protein as compared to the DNA prime would further increase the likelihood of targeting constant regions of Env. The results showed that high titres of heterologous HIV-1 neutralizing antibodies were induced against Tier 1 viruses in the TZM-Bl assay and against Tier 2 viruses in the A3R5.7 assay. These data support further exploration of such heterologous regimens for induction of broadly neutralizing antibodies.

Previous work performed by Andrea Cara at the Instituto Superiore di Sanità has shown that integrase defective lentiviral vectors (IDLV) expressing HIV-1 gp120 can be used for the induction of polyfunctional CD8+ T-cell responses and gp120-specific serum antibodies in mice [18]. In addition, *ex vivo* transduction of human macrophages and DC with IDLV expressing the influenza matrix protein M1 induced expansion of antigen-specific polyfunctional CD8+ T-cells. At the EUROPRISE meeting, Cara presented new data demonstrating that the addition of the SIV VpX protein to IDLV vectors enhances transduction efficiency in human and simian DC, thereby improving antigen presentation. Recent studies have demonstrated that host cell SAMHD1 acts as an HIV-1 restriction factor by inhibiting viral DNA synthesis [19]. The SIV VpX protein targets molecules in SAMHD1 to the proteasome for degradation, thereby enabling viral replication. Thus, by inhibiting SAMHD1-mediated restriction of viral replication, the SIV VpX protein may enhance the transduction efficiency of IDLV in human and simian DCs, thereby admitting the use of lower doses of vaccine, which improves the safety profiles for IDLV. Other studies of retroviral vector use were performed by Pepe Alcami et al. from Instituto de Salud Carlos III, Madrid. They demonstrated that RT-defective virions acted as effective immunogens for T-cells.

Rico Blochmann, a young investigator from the Robert Koch Institute, described the development of novel replicating foamy virus vectors capable of prolonged antigen presentation. Infection with low-level replicating retroviral foamy virus is often symptom-free, and the viral particle could be used as an alternative vector in an HIV vaccine construct. A foamy hybrid virus has already been tested in hamsters, with rapid responses to encoded proteins, and a vector for rhesus macaques has been developed. Two reading frames of the viral genome were successfully removed without affecting viral replication in the macaque model. A viral vector expressing GFP and a variety of HIV CTL epitopes have been developed and are being tested *in vitro*.

A new strategy for genetic HIV vaccination based on heterologous prime-boost regimes using different vectors and viral genes was presented by Marc Reudelsterz from the Robert Koch Institute. This approach, designed to avoid a decrease in vaccine efficacy following repeated vaccination using the same vector, was adopted in parallel for HIV- and SIV vaccines. Codon-optimised constructs (*rev-nef-tat-gag* and *pol*) were used to prepare vaccine vectors based on plasmid DNA, recombinant adeno-associated virus (rAAV) and recombinant adenovirus 5 (rAd5). The magnitude and breadth of T-cell responses were increased following heterologous triple-immunization of mice with DNA-, rAd- and rAAV-based immunogens, especially against the Pol and Gag gene products of HIV and SIV.

An innovative method for HIV vaccine delivery was presented by the EUROPRISE student Charlotte Pollard from the Institute of Tropical Medicine in Belgium. She has delivered Gag mRNA complexed to DOTAP/DOPE, a cationic lipid used for transfection subcutaneously in mice to be processed and presented by immune cells (unpublished). In an *in vivo* killing assay, Gag-expressing splenocytes were shown to be selectively killed. When combined with a Gag mRNA - p24 protein boost, both IgG1 and IgG2 antibodies were detected, as well as Th1 and Th2 responses. In addition, inflammatory DCs were detected in lymph nodes one day after vaccination. Other means to enhance transfection consist of electroporation of DNA- or RNA-based antigens significantly raises immunogenicity of the expressed protein [20].

## Microbicides & Pre-exposure prophylaxis (PrEP)

### Clinical studies

Development of an effective microbicide against HIV-1 continues to be a major target for the HIV community. The microbicide session of the Europrise meeting gave a comprehensive update on preclinical and clinical studies of novel potential microbicides.

**Figure 1:**
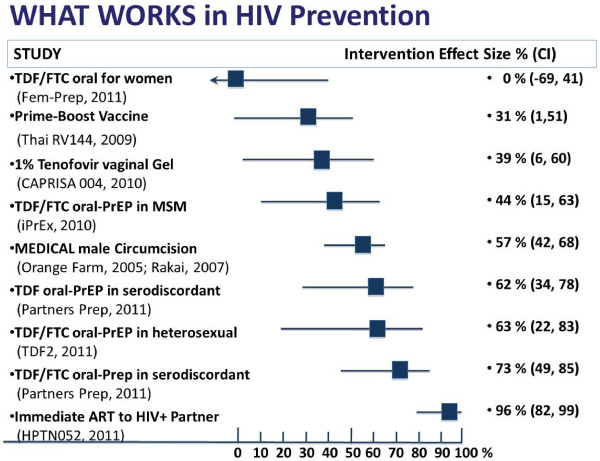
**Reduction of HIV transmission by selected biological or drug interventions.** Modified from Shattock et al [34] and expanded by us.

Ian McGowan (University of Pittsburgh) discussed microbicide development in the context of the encouraging CAPRISA 004 microbicide efficacy trial [21] and provided a comprehensive review of the NIH-funded Microbicide Trials Network's clinical trial portfolio (see Figure 1).

Results from the CAPRISA 004 trial of tenofovir 1% gel and other trials of oral pre-exposure prophylaxis (PrEP) of tenofovir support the topical and oral use of antiretrovirals for prevention of HIV infection. Differences in protection rates across these clinical trials may be explained by a variety of factors, including the heterogeneity of study populations, the route and frequency of dosing, as well as differential levels of tenofovir metabolites in the vagina compared to the rectum after oral dosing, and much higher levels of active drug in the genital tract after topical antiretroviral treatment. Although the precise threshold of drug required for protection has yet to be determined, it was shown that topical administration achieved the highest levels of drug in the genital tract and low systemic levels.

A major driver of overall effectiveness of either microbicides or oral PrEP was adherence; this should be taken into account in the development of future trials and microbicide delivery strategies. Besides improving the methods for collecting information on product use, priority should now be given to establishing the safety of tenofovir gel in pregnancy, adolescence, and rectal use.

Tenofovir as pre-exposure prophylaxis (PrEP) has been shown to significantly reduce HIV incidence, alone or in combination with emtricitabine. Whereas the CAPRISA microbicide trial observed a 39% reduction in HIV acquisition with a ‘before and after’ sex schedule of tenofovir vaginal gel [21] and the iPREX PrEP trial observed a 44% reduction with daily oral Truvada (tenofovir/emtricitabine), the FEM-PrEP trial showed no effect for Truvada in women [22]. In May 2011, several researchers came together in a EUROPRISE workshop to better understand how adherence and biology explained the diverse effectiveness in these three trials. The most likely biological hypothesis was that when drug was administered orally, the level of active drug in the cervico-vaginal tissue was influenced by endogenous and exogenous female hormones. This question is now being investigated in a clinical trial. The promise of antiretroviral-based prevention also inspired a satellite meeting co-organised by AVAC, EATG and the Forum for Collaborative HIV Research during the 6th IAS Conference on HIV Pathogenesis, Treatment and Prevention (July 2011 in Rome). This meeting concluded that it was time for the development of a multi-stakeholder consensus statement on the need to expand efforts to use antiretroviral-based combination prevention to achieve community-level control of HIV infection. Today we have far more options for preventing HIV than when EUROPRISE started (Figure 1) but considerably more research will be required before we know how best to deploy these alone and in combination in public health programmes.

### New components for a microbicide

Neutralizing antibodies have been considered as novel microbicide candidates, since antibodies directed against conserved regions of HIV-1 proteins may inhibit HIV entry into host cells. Llama heavy-chain antibody fragments (VHH) are a class of molecules recently described as potent cross-clade HIV-1 entry inhibitors. Laura McCoy et al. (University College of London) showed that VHH antibodies isolated from libraries derived from llamas immunized with recombinant gp120 or trimeric gp140, have neutralizing activity against several strains of HIV-1. In particular, one potential candidate neutralizing antibody, J3, was identified on the basis of its capacity to neutralize the majority of HIV-1 pseudoviruses tested. Moreover, the importance of llama antibodies was underlined by their characteristic of being chemically and thermally stable and by their low production cost. Feasibility studies are now needed to determine the developmental pathways needed to bring this approach to clinical trials.

The EU funded Pharma-Planta Integrated Project was presented by Julian Ma (St George´s, University of London) as a potential platform for large-scale production of anti-HIV neutralizing antibodies produced in transgenic tobacco plants. He stressed the fact that this approach represents a new affordable manufacturing platform for proteins and microbicide combinations. Indeed, following cGMP approval for production of antibodies in plants, a clinical trial application has been approved, allowing a Phase I clinical trial to start. The results of the trial are anticipated to be available in late 2012.

Reverse transcriptase (RT) inhibitors might also form important components of an effective microbicide, as demonstrated by the CAPRISA 004 trial of tenofovir gel. TMC-120, a Non-nucleoside Reverse Transcriptase Inhibitor (NNRTI), is currently being evaluated in a phase III efficacy trial, while other NNRTIs are being tested in preclinical trials. Susan Fetherston et al. (Queen’s University, Belfast) presented data on MC1220, another NNRTI evaluated in a non-human primate challenge study. MC1220 has previously been demonstrated to have potent anti-HIV activity in vitro and partial efficacy when formulated as a liposomal gel in macaques exposed to RT-SHIV [23]. Dr Fetherston presented results on the use of MC1220-loaded vaginal rings in macaques and showed the presence of detectable drug levels in both vaginal fluid and plasma during the entire period of ring-placement. Drug release from vaginal rings was also demonstrated in *in vitro* experiments using simulated vaginal fluid. After the PK study (days 0-28), the same macaques were included in a challenge study. A one-week washout period was followed by placement of either a placebo (n = 4) or a 400 mg MC1220 ring (n = 6) on Day 35. Macaques were subsequently challenged with vaginally-administered RT-SHIV162P3 once a week for four weeks, on days 42, 49, 56 and 63. In the placebo arm, 1 of 4 macaques was infected within two weeks of placebo ring placement and following a single vaginal challenge; the remaining three macaques in the placebo arm were infected within four weeks of ring placement and following three challenges. The active ring provided a greater degree of protection, with one macaque infected by week 4, another by week 5, and a further two by week 6. At the end of six weeks, and following 4 challenges, 2/6 macaques remained uninfected. These results make MC1220 a promising compound for further development as a topical microbicide, and vaginal rings a promising device for delivering microbicides.

The ultimate goal for prevention is to increase the efficacy of individual strategies, providing a comprehensive blockade to HIV infection. Studies are underway to determine whether the potential protection delivered by microbicides could be increased by combining them with vaccination. Such a concept is at the heart of the Europrise Network of Excellence. Roger Le Grand et al. (CEA, Paris) presented preliminary data on a study performed in Cynomologus macaques where animals were immunized intranasally with trimeric gp140 subtype B and C proteins, followed by two intramuscular boosting immunizations with the same two proteins formulated in MF59. Animals were then challenged intravaginally in the presence or absence of 1% Tenofovir microbicide gel. The immunization schedule induced good systemic and vaginal immunity as measured by specific IgA and IgG levels, and presence of neutralizing antibody to tier 1 viruses in the serum but not in the vaginal secretions. The combination of vaccine with microbicide showed enhanced protection against SHIV challenge when compared to animals treated with microbicide alone; both microbicide alone and the combination provided significant protection against low-dose repeat viral challenge when compared to naïve challenged animals. Ongoing studies will assess the efficacy of the vaccine alone.

## Mucosa and HIV-1/SIV transmission

### Mucosal biology and viral infection

The potential influence of the human vaginal microenvironment on HIV transmission was addressed by Jordan Kyongo et al. (Institute of Tropical Medicine, Antwerp, Belgium). The presence of soluble immunomodulatory factors was evaluated in the genital fluids of a cohort of healthy Caucasian women. It was shown that levels of IL-1α, IL-1ra, IP-10, MIP-1b and the antimicrobial protein β-defensin fluctuated during different phases of the menstrual cycle. Such fluctuation may modulate susceptibility to HIV infection, potentially an important consideration when designing vaginal intervention strategies.

In contrast, Kevin Mendonca, a student from the University of Siena, demonstrated the importance of the seminal compound spermine in down-regulating TLR expression on vaginal epithelia cells. He raised the possibility that as activation of some TLRs, for example TLR-3, significantly reduces viral infection, the action of spermine might be a factor that influences male-to-female transmission of HIV through dampening of innate antiviral responses. However, the *in vivo* relevance of these findings requires further investigation.

Mariangela Cavarelli from San Raffaele Scientific Institute in Milan discussed studies concerning the susceptibility of dendritic cells and CD4+ T lymphocytes to HIV-1 infection using an *in vitro* model of intestinal mucosa. The influence of epithelial cells on HIV-1 infection of DCs and CD4+ T-cells was investigated to provide a better understanding of virus transmission events at the intestinal mucosa. Supernatants from the epithelial cell line Caco-2 were used to condition monocyte-derived DCs and CD4+ T-cells, and the expression of HIV co-receptors as well as HIV infection were analyzed. The infectibility of DCs with R5 or X4 virus was altered in terms of a reduced integration of viral genome. Both R5 and X4 viruses infected both conditioned and non-conditioned CD4+ T-cells better than DCs, although conditioning did not alter CD4+ T-cell susceptibility to HIV-1 infection.

Several studies have shown that infected seminal leucocytes can cross intact vaginal epithelia to reach the submucosa and the draining inguinal lymph nodes. In this way, cell-associated virus may still establish infection by escaping the high microbicide concentrations in the vaginal lumen. However, virus budding from these migrating leucocytes could be attenuated by prior vaginal drug exposure. Katrijn Grupping and Philippe Selhorst (ITM, Antwerp, Belgium) reported that, in contrast to other antiretrovirals, pre-treatment of HIV-infected PBMCs with M48U1 CD4-mimetic results in a dramatic and prolonged reduction of virion infectivity. This ‘memory effect’ is related to gp120 shedding from membrane-embedded HIV envelope proteins. Inclusion of M48U1 in topical microbicides may therefore help to reduce the risk of systemic infection by infected cells.

Another EUROPRISE-supported student, Kelly da Costa from St George’s Hospital in London, presented research on *in vitro* infection of virus-naïve macaque colorectal explants. She compared SIV superinfection of explants taken from SIV-infected macaques with either disease progressor or non-progressor phenotype. Colorectal tissues from progressors did not show extra virus production after superinfection challenge, whereas the pattern after challenge of tissues from LTNPs was more variable. While both IgG and IgA total immunoglobulins were secreted from the explanted cultures of SIV-infected macaques, only IgG gp130-specific antibody was detected.

Elisa Saba from the San Raffaele Scientific Institute presented data concerning factors affecting HIV-1 permissibility in a human cervical explant model. The most prominent CD4+ T-cell type in the mucosa was of the effector memory phenotype, and 50% and 80% of the cells expressed CCR5 and CXCR4, respectively. R5 virus efficiently infected the explants over time, while X4 only infected 4/27 donors, despite the high levels of CD4 and CXCR4 in the tissue. Saba also showed that the most permissive donors had high progesterone blood levels, which is in agreement with the literature, suggesting that women on contraceptive pills containing progesterone may be more easily infected than menopausal women. These data support the use of tissue explant models to further characterize factors of importance for the sexual transmission of HIV.

## Mucosal vaccination

Given that most HIV-1 transmissions occur during sexual exposure of the genito/rectal mucosa, an ideal HIV-1 vaccine candidate should induce protective antibody responses at the mucosal surfaces [24]. Although some studies have shown that systemic delivery of HIV-1 vaccines can induce HIV-specific immune responses at the mucosa [25,26], findings in mouse models indicate that systemic delivery of HIV-1 vaccines can compromise the quality or avidity of the HIV-specific immune responses at mucosal sites [27].

Paul McKay (Imperial College in London) and co-workers from Queens University (Belfast) have developed a novel antigen delivery device to induce antigen-specific immune responses directly at the vaginal mucosa. A silicone elastomer ring carrier was used to deliver recombinant gp140 antigen adjuvanted with a resiquimod (R848), a TLR 7/8 agonist, into the vaginal cavity of sheep. Antibody responses in sheep receiving only the ring devices were compared to antibody responses in sheep primed intramuscularly with gp140 and R848 followed by insertion of the antigen loaded rings. Results showed that both systemic and vaginal priming induced antigen-specific IgG and IgA responses in serum and vaginal fluid, with the levels of IgA being 30-fold higher at the mucosal surfaces than in serum. It was observed that intramuscular injection did not prime for the development of a mucosal IgA response. Taken together, these data favour the use of this novel ring device for the induction of antigen-specific systemic and mucosal humoral immune responses.

Linda Klavinskis (Kings College, London) described an alternative route of immunization designed to induce mucosal cellular responses. Air-dried dissolvable micro-needle arrays were developed to specifically target dendritic cells in the skin, thereby enhancing antigen presentation. Microneedle delivery of a rAd5 vector encoding HIV-1 Gag induced antigen-specific CD4+ and CD8+ T-cell responses in mice, and these responses were as efficient as those induced by subcutaneous or intradermal vaccination. Importantly, the use of this micro-needle array device on the skin allowed the induction of Gag-specific CTL responses in the female genital mucosa. Experiments using transgenic mice with dendritic cells expressing langerin-DTReGFP or CD11c-DTReGFP, demonstrated that conventional CD11c^high^ dermal DCs, but not epidermal Langerhans cells are required for the induction of CD8+ T-cell responses by micro-needle array delivery.

The impact of intranasal immunization on the distribution of antigen-loaded antigen-presenting cells (APCs) and of primed antigen-specific T-cells was studied by Medaglini et al. (University of Siena). Nasal immunization elicited primed T-cells not only in draining lymph nodes but also in non-draining lymphoid sites. They showed, through the use of Fingolimod, a drug that causes lymphocyte sequestration within lymph nodes, that the presence of primed T-cells at distal lymph nodes was due to migration of locally primed T-cells. In contrast to primed T-cells, antigen-loaded APCs could only be detected in the draining mediastinal lymph nodes, and no antigen-loaded APCs were observed in distal lymph nodes. T-cell entry into iliac lymph nodes was found to be CD62L dependent, while CD62L and α4β7 were responsible for homing to the mesenteric lymph nodes. Furthermore, they showed that nasal boosting induces IL-2 producing T-cells, antigen-specific IgG and IgA production and antigen-specific B memory lymphocytes. To further characterise T-cell proliferation, mathematical models were used and it was observed that following nasal immunization, the probability of T-cells entering division is higher and the time of the first division is faster compared to vaginal immunization. Together, these results provide additional information on mucosal vaccination strategies.

The priming of T-cells is a critical event that influences the type and magnitude of immune responses. Further data from Medaglini’s group, presented by Fabio Fiorino, evaluated primary CD4+ and CD8+ T-cell activation induced by different mucosal routes of immunization. Mice vaginally and nasally immunized with OVA + CpG were studied for clonal expansion, activation and migration markers at different time points. Both vaginal and nasal routes of immunization induced high T-cell clonal expansion in the respective draining lymph nodes by day three after vaccination, whilst at day 5, primed T-cells were also detected in distal lymph nodes and in the spleen, with higher percentages detected following the nasal route immunization. The proliferating T-cells were activated and acquired migration properties as indicated by the modulation of CD44, CD45RB and CD62L.

## Neutralizing antibodies

HIV-2 is a somewhat understudied virus with a lower pathogenicity and transmission rate than HIV-1. Gulsen Özkaya Sahin and Marianne Jansson from Lund University in Sweden demonstrated that the breadth and potency of neutralizing activity in plasma differ between HIV-1 and HIV-2 infected individuals. Plasma from HIV-2 patients was shown to neutralize HIV-2 isolates at a high frequency, whereas plasma from HIV-1 patients had much lower intratype neutralizing activity against HIV-1 isolates. The intratype neutralizing activity correlated with viral load in patients infected with HIV-1 but not with HIV-2. Hence the neutralizing activity in HIV-2 infection does not seem to require high levels of antigen stimulation, whereas this is more important in HIV-1 infection. Jansson concluded that the viral structure of HIV-2 may promote a more potent humoral immune response and/or HIV-2 itself may be more neutralization sensitive than HIV-1.

Stefania Dispinseri (San Raffaele Scientific Institute) presented data from paediatric infections, correlating disease progression with neutralizing responses. HIV-1 infected children with slow disease progression developed an increasing breadth of neutralizing response over time, whereas fast-progressing children did not.

A similar study, conducted by Katharina Raue, a student from the German Primate Center, analysed the immune responses of infected rhesus monkeys, grouped into controllers or progressors. Animals controlling the infection demonstrated higher immune response than progressors, both humoral and cellular, and the response was directed against Gag proteins and not Envelope.

Not all patients develop broadly neutralizing antibodies; in fact, only 10-30% of HIV-1 infected patients possess antibodies with a strong cross-reactive neutralizing activity and less than 1% have broadly neutralizing antibodies. Zelda Euler from the University of Amsterdam performed a genome-wide association study, to learn more about which genetic markers are involved in the development of antibodies with broadly neutralizing activity. The study included 335 HIV-infected men who have sex with men from the Amsterdam Cohort with known neutralizing activity of their sera. Single nucleotide polymorphisms (SNP), in the major histocompatibility complex gene region, close to MICA and HCP5 genes on chromosome 6, were found to associate with broadly neutralizing activity. HCP5 encodes HLA complex protein 5, and has previously been associated with viral load and disease progression in HIV infected individuals [28,29]. None of the identified SNPs which were associated with broadly neutralizing activity were associated with the clinical course of disease progression. In addition, the association between SNPs or broadly neutralizing activity of antibodies was not related to viral load or CD4+ T-cell count at viral load set-point. Although this finding is remarkable, its application to our understanding of the factors modulating the mounting of an effective immune response is not yet clear.

Marie Borggren, from the University of Lund, compared how CCR5 and CxCR4-using HIV-1 evolve during end-stage disease and how the immune responses correlate with the evolution. The Env proteins of end-stage viruses were often more positively charged and had fewer glycans than virus from the chronic stage of disease. End-stage R5 viruses were shown to use the *trans*-infection route from DC to T-cells less efficiently, which might be due to loss of a specific glycan in gp120.

David Reinhart (Polymun and University of Natural Resources and Life Sciences, Vienna) discussed the switching of the isotypes of anti-HIV-1 IgG antibodies 3D6 and 4B3 recognizing the ectodomain of gp41 to an IgA isotype and their expression in CHO cells in serum-free medium as dimeric IgA. The purification scheme mainly relies on IgA affinity chromatography, using VHH ligands. Unlike previously published protocols, the purification schedule can be used for any IgA irrespective of alpha heavy chains, light chains and their subtypes [30]. These new reagents will allow the study of the influence of antibody isotype on their function with respect to antiviral activity against HIV. Studies are pending to investigate the potential role of IgA during viral exposure at the mucosa.

Enas Sheik-Khalil (University of Lund) described the development of an image-based high-throughput as well as high-content HIV neutralization assay. The assay is based on a plaque reduction assay along with a specialized image analysis tool tailored to analyse neutralization. An additional parameter, mean plaque area, enables the detection of virus fusogenicity to characterize the duration in vitro of neutralization. A decreased plaque size may indicate avidity of neutralizing antibodies. The assay demonstrated equal or higher sensitivity compared to conventional neutralization assays.

### Eliciting antibodies

Eliciting new type of antibodies and defining novel epitopes will be critical for future vaccine development. Mark Hassell et al. (NIBSC) have produced novel C clade specific antibodies by priming mice with plasmid DNA encoding either CN54 or ZM96 gp140 genes, followed by a CN54 trimeric gp140 boost, all of subtype C. Applying the trimeric immunogens, 18 HIV-specific antibodies were successfully generated. However, no novel epitopes were defined. Although all isolated antibodies bound CN54, none were able to recognise and neutralise epitopes that are specific for the trimeric envelope structure.

Another approach to elicit novel antibodies, presented by Pierpaolo Racchiolli et al. (University of Verona), established new anti-HIV-1 antibodies by immunizing mice with CHO cells expressing gp120/gp41 and CHO cells expressing CD4 and CCR5 to generate fusion intermediate structures of the HIV envelope. One hybridoma clone, 10 F12, which showed the highest HIV-1 neutralization activity and lowest cell reactivity, was further investigated and stably produced in CHO cells as a chimeric antibody. Hybridoma and chimeric antibodies both neutralized several HIV viruses.

### Other roles for antibodies

HIV infection can be inhibited by both neutralizing and non-neutralizing antibodies via the Fc receptor. Infection of myeloid dendritic cells, mDC, has been shown to be inhibited using both types of antibody. In contrast, Alexandre Lederle, a student from University of Strasbourg, demonstrated that infection of plasmacytoid dendritic cells, pDC, can be inhibited only by neutralizing antibodies, not by non-neutralizing antibodies, despite the expression of Fc receptors on the surface of pDC.

As discussed previously, transmission of HIV-1 via mucosal tissues during sexual intercourse globally represents the main route of infection. Immature dendritic cells reside in the genital mucosal tissues and are believed to be among the first cells to be in contact with HIV. An infected DC may act as a “Trojan horse” for HIV and further transmit viruses to primary CD4+ T lymphocytes. Antibodies that inhibit such viral transfer could prevent HIV infection. Bin Su from the University of Strasbourg addressed this hypothesis and demonstrated that neutralizing antibodies could efficiently inhibit the transfer of several R5 HIV-1 strains from primary DCs to autologous CD4+ T lymphocytes. Furthermore, he showed that neutralizing as well as non-neutralizing antibodies could decrease HIV-1 replication in DCs. Induction of both types of antibodies directly at mucosal sites may thus be beneficial when designing a future vaccine.

Besides neutralization, HIV-specific antibodies could thus have an impact on protection against HIV-1 transmission in several ways. Such HIV-specific antibodies could target not only free virions, but also infected cells, as well as the HIV transfer from infected cells to other cells. In order to understand the contribution of antibodies induced against these targets and especially the role of Fc-mediated protection, Marina Biedma from the University of Strasbourg investigated several inhibitory functions of anti-HIV IgGs. It was found that non-neutralizing inhibitory antibodies (NNIAbs) could mediate inhibitory activities such as ADCC or phagocytosis. Indeed, it was demonstrated that NNIAbs such as 4B3 and 246D were highly efficient in capturing free virions when compared to neutralizing antibody 2G12, 4E10 or 2F5. Furthermore, NNIAbs 4B3 and 246D revealed Fc-mediated inhibitory activities on viral replication in macrophages and DCs similar to some neutralizing antibody. When applied locally at the vaginal site of macaques, these NNIAbs were able to significantly decrease the peak viral load following vaginal experimental challenge with SHIV SF162P3. These results suggest that NNIAbs may be important in controlling viral infections.

In addition to broadly neutralizing antibodies, other targets for antibodies with alternative antiviral function are being explored. Donato Zipeto from the University of Verona described the identification of one such new target by unravelling the association between HLA-C and gp120. Using bimolecular fluorescence complementation analysis, it was shown that HLA-C competes for binding between β_2_m and gp120. Furthermore, association of gp120 with HLA-C happens early in the endoplasmic reticulum and continues during passage through the Golgi apparatus and early endosomes, until presentation at the cell membrane. It was found that HLA-C specifically incorporates into HIV-1 envelopes and increases viral infectivity by increasing viral envelope fusion with R5 and X4 HIV strains. A compound or antibody that could disturb this association might decrease viral infectivity. Furthermore, antibody to HLA-C displayed on the virusor virus-infected cell surface might participate in ADCC.

## Innate immunity against HIV

In a setting where ongoing attempts to develop an HIV-1 vaccine face significant challenges, investigation of innate immune factors and the host response to infection are crucial for the development of novel prophylactic strategies against HIV/AIDS. Innate responses not only influence mucosal transmission and establishment of initial infection foci, but may also have an impact on the subsequent level of ongoing viral replication and rate of disease progression [31]. Studies were presented to explain the contributions made by components of the host innate response to HIV acquisition and spread versus its control.

Magarita Bofill (IrsiCaixa, Barcelona) has previously described that co-stimulation of mononcytes with IL-12 and IL-18 triggers the survival and differentiation of monocytes to macrophages, important players in innate immunity [32]. As an advance on this work she demonstrated that the treatment of differentiated macrophages but not monocytes with IL-12 and IL-18 induces production of IFN-γ in an antigen-independent manner. Absence of proviral or integrated DNA in target macrophages identified the inhibitory effects of IL-12 and IL-18 as entry events. Furthermore, IL-12 and IL-18 induced Apobec3G up-regulation in these target cells.

Interferon (IFN)-induced intracellular antiviral proteins, referred to as restriction factors, are capable of interfering with retroviral replication at various steps of the viral life-cycle. Elisa Vicenzi’s group (San Raffaele Scientific Institute) has exploited cell clones isolated from the U937 promonocytic cell line, either permissive or non-permissive for HIV-1 replication. Data revealed that among known HIV-1 restriction factors, the IFN-inducible Tripartite motif-containing protein 22 (TRIM22) was the only factor constitutively expressed in non-permissive but absent in permissive U937 cells. Conversely, stable knock-down of TRIM22 rescued the non-permissive cell line, while overexpression of TRIM22 in a permissive cell line inhibited HIV-1 replication. A similar inhibitory effect of TRIM22 was confirmed in human A301 T-cells. Most likely, nuclear TRIM22 impairs HIV-LTR driven transcription in a Tat- and NF-κB-independent manner [33].

## Adjuvants

Specific adjuvant formulations triggering innate immune signalling pathways can improve the potency and quality of adaptive immune responses. Therefore, many studies are focusing on the development of novel adjuvants that might increase the immunogenicity of HIV-1 antigens.

Annette Sköld (Karolinska Institutet) presented a poster on the use of single-stranded DNA oligonucleotides (ssDNA ODNs) as an inhibitor of detrimental TLR3 responses. Results showed that the addition of ssDNA ODNs inhibited the TLR3-triggered cytokine release and maturation of human DCs. In addition, ssDNA ODNs blocked TLR3-mediated release of pro-inflammatory cytokines in the airways of cynomolgus macaques. In summary, while the use of ssDNA ODN might open novel perspectives for clinical treatment of TLR3-mediated inflammatory disorders, it was clear that combining ssDNA ODN with TLR3 agonists may be detrimental for the efficient induction of immune responses.

In work presented by Quentin Sattentau (University of Oxford), the nucleic acid transfection reagent polyethyleneimine (PEI) was shown to exhibit potent mucosal adjuvant properties. Intranasal administration of the influenza HA antigen, adjuvanted with PEI, protected mice against viral infection and disease. Moreover, in the induction of protective responses, PEI was superior to other known mucosal adjuvants, including CpG. Similar results were obtained for herpes simplex virus 2 (HSV-2) gD and HIV-1 gp140 antigens. It was shown that PEI forms complexes with antigens, thereby targeting DCs. Indeed, such particulate antigen systems are similar in size to microorganisms, and are consequently efficiently taken up by phagocytic cells, including DCs. In addition, PEI was shown to activate innate immune responses, including inflammasome- and MyD88-dependent pathways.

## HIV/SIV pathogenesis and disease progression

### Correlates of pathogenicity

Based on previous observations that a high frequency of adenosine nucleotide in HIV/SIV RNA correlates with the pathogenicity of a lentivirus, a study presented by Frederic Tangy (Institut Pasteur) focused on the effect of the viral A/C/G/T nucleotide ratio on stimulation of innate immune responses. Synthetic SIVs genomes were designed without modifying regulatory elements or sequences of amino acids. The first SIV was subjected to codon changes of *gag* and *pol* genomic regions, which resulted in a dramatic decrease in replicative capacity. The second SIV harboured only a *pol* optimized gene, which resulted in a reduced ability to stimulate type-I interferon *in vitro* in cellular assays. Nucleotide patterns (without changed amino acid patterns) may thus play a role in the induction of innate immunity and in virulence.

A EUROPRISE student Aneela Javed (German Primate Center) investigated factors correlated with CD8+ T-cell Non-Cytolytic Antiviral Activity (NCAA) in SIV-infected rhesus macaques. NCAA is associated with reduced transmission of HIV and slow disease progression. NCAA + and NCAA- animals were identified using an in vitro viral inhibition test. Differentially expressed genes in CD8+ T-cells from NCAA + and NCAA- animals were investigated using microarrays. FAM26F, translating a membrane-bound protein with undefined function, was identified as a candidate gene. Furthermore, the expression of FAM26F is negatively correlated with viral load, while positively correlated with the expression of two cytokines, IP-10 and MX. Further studies in humans are ongoing.

### T-cells

Not all individuals have the same T-cell profile, and co-infection with HIV and other viruses, bacteria or parasites may influence the target T-cell pool for HIV. Understanding the co-infection responses and the consequence for the HIV infection could affect individualized treatment. William Paxton from the University of Amsterdam compared the *mycobacterium tuberculosis* (MTB)- and cytomegalovirus (CMV)-specific CD4 T-cell responses in patients co-infected with HIV. His results demonstrate that the CD4 T-cell population is skewed in these patients, with a persistent CMV-specific T-cell response but a MTB-specific response that was rapidly depleted. The CMV-specific CD4 T-cells had a mature phenotype, with high MIP-1β and low IL-2 producing cells. In contrast, the MTB-specific CD4 T-cells were less mature, with more IL-2 production and less MIP-1β. These findings confirm that HIV preferentially infects IL-2 producing T-cells, and that MIP-1β producing cells are more resistant.

### B-cells

Nicolas Ruffin from the Karolinska Institutet investigated the effect of HIV infection on the B-cell compartment of HIV-infected patients. He found that the levels of sCD14, a marker of microbial translocation, correlated with B-cell activation and loss of resting memory B-cells. sCD14 was associated with higher IL-21R expression on resting memory B-cells and the B-cells bearing IL-21R were shown to be more susceptible to apoptosis. This finding provides an important insight into how the humoral arm of the immune system may be affected by the microbial translocation that occurs during HIV-1 infection.

Simone Pensieroso (San Raffaele Scientific Institute) examined the hypothesis that highly efficient long-term antiretroviral therapy could quantitatively restore all B-cell subsets. In particular he examined resting memory B-cells, which are responsible for secondary immune responses and are severely depleted in HIV-infection. After failure of previous therapies, 30 HIV-1 infected multi-drug experienced patients were treated with Highly Active Antiretroviral Therapy (HAART including Raltegravir) and followed for 144 weeks. 72 age-matched uninfected individuals were used as healthy controls. An effective Raltegravir-including regimen was shown to restore all B-cell subpopulations with the exception of the resting memory B-cell subset when monitored for up to three years. A longer follow-up study might determine whether resting memory B-cells need even more time to recover.

## New methods and reagents

### Measurement of immune responses

One of the objectives of the EUROPRISE consortium has always been to develop new techniques, assays and standard reagents and make them available to researchers around the world to help accelerate research on HIV vaccines and microbicides.

A new assay developed by Mabtech with support from EUROPRISE is the FluoroSpot assay, which is based on the same principle as the well-known ELISpot assay. The FluoroSpot assay is characterized by high sensitivity and specificity and can be used to analyse simultaneous secretion of cytokines by T-cells. It also allows the quantification of singly and dually secreting cells. Clearly, this can provide important information about the quality of a specific T-cell response. So far, combinations of IFN-γ /IL-2, IFN-γ /IL-5, IFN-γ /IL-13, and IFN-γ /IL-17A have been developed. Furthermore, it is possible to detect IgG and IgA secreted by B-cells in a single well and analysis can be performed on mice, monkey and human cells.

JPT Peptide Technology, a company in Germany, presented their technology platform for peptide microarrays. They have generated a versatile peptide library spanning the immunogenic fractions of the HIV proteome with high coverage across viral clades. JPT have been successful in generating overlapping peptide sets representing all immunogenic regions of target HIV proteins. The final library of 5572 different 15-mer peptides achieves coverage of 86% of all 3578 M-group and recombinant sequences of p24 as well as coverage of 50% of the published 2248 sequences of gp160. A major advantage of this library design is the ability to condense and cover the immense complexity of the immunogenic domains of the HIV proteome with only 5572 overlapping peptides that can be accommodated on a single high-density peptide microarray for clinical HIV research. These peptide microarrays have several applications, such as biomarker identification, vaccine target identification, immuno-monitoring, mapping B-cell responses, and profiling of enzymatic activity. Monitoring the antibody response in detail before and after vaccination in patient samples is crucial. In this context, the peptide microarray, which gives insights into the antigen-antibody interaction on the sub-protein level, may complement other assays which rely on the entire antigen. Nevertheless, the technology is restricted to the extent that it can map only linear and not conformational epitopes.

### Reagents

The Division of Retrovirology at the NIBSC in the UK produces a variety of biological standards and reference materials designed as quality controls for HIV diagnostic (serological) assays and nucleic acid-based amplification techniques. The HIV-1 and HIV-2 Internal standards (IS) developed by NIBSC have been used to standardize both commercial and in-house assays. NIBSC has also developed a first genotype panel containing genotypes A-H, as well as HIV-1 working reagents calibrated against internal standards to support serology-based diagnostic assays. A selection of recently transmitted subtypes and subgroups of HIV-1 strains has been assembled to create a panel with a great variety of viral stocks. The viral isolates were collected from HIV-1 infected patients and expanded in PHA stimulated PBMCs, whereafter they were characterized using RT-PCR and phylogeny. The stocks are held by NIBSC and are available to all HIV researchers.

The Center For AIDS Reagents (CFAR) at NIBSC has been able to support HIV/AIDS research through the supply of reagents to scientists in Europe and worldwide. With the help of 173 novel materials provided by the EUROPRISE collaborators, CFAR now has a repository of over 6000 reagents. They have also successfully produced novel, lyophilized cell preparations for detecting and quantifying CMI assays. The availability of such useful materials is a legacy of success for the EUROPRISE group.

## Concluding remarks

The fifth EUROPRISE Network annual conference was held in Prague in November 2011 in an atmosphere of optimism. Imaginative and novel strategies to be used in HIV prophylaxis and intervention were discussed.

The scientists involved in this Network of Excellence have participated in over 200 multi-author papers, weekly bulletins have been provided within and outside the network, and HIV/AIDS reagents have been provided to colleagues worldwide. The Network´s extremely successful students have bound the research groups together, formed networks of their own and brought an immeasurable feeling of collaboration, sharing of ideas and positive aspects of future research collaboration to the Network. Such integrated science will permit continued developmental research from discovery to clinical trials, some of which are described in this review.

The co-usage of vaccines and microbicides is unique; the Network has brought forward research in this area and is now also starting preclinical and clinical trials with the aim of combining the two. The meeting in Prague was focused on collaborative work between partners, in particular scientific work performed and presented by the Network’s PhD students. The abilities of students to perform and present up-to-date science promise a bright future for the next generation of HIV researchers in Europe.

## Competing interests

The authors declare that they have no competing interests.

## Authors’ contributions

NR, MB, ZE, FF, KG, DH, AJ, KM, CP, DR, ES, ESK, AS and SZ were in charge of the writing of dedicated chapters covering the various sessions of the conference. RS, GS, FG and BW organized the sessions and the writing, and corrected and revised the manuscript. All authors read and approved the final manuscript.

## References

[B1] Brinckmann S da Costa K van Gils MJ Hallengard D Klein K Madeira L Mainetti L Palma P Raue K Reinhart D Rational design of HIV vaccines and microbicides: report of the EUROPRISE network annual conference 2010 J Transl Med 2011 9 40 10.1186/1479-5876-9-40 21486446 PMC3086860

[B2] Wahren B Biswas P Borggren M Coleman A Da Costa K De Haes W Dieltjens T Dispinseri S Grupping K Hallengard D Rational design of HIV vaccine and microbicides: report of the EUROPRISE annual conference J Transl Med 2010 8 72 10.1186/1479-5876-8-72 20659333 PMC2922088

[B3] Rerks-Ngarm S Pitisuttithum P Nitayaphan S Kaewkungwal J Chiu J Paris R Premsri N Namwat C de Souza M Adams E Vaccination with ALVAC and AIDSVAX to prevent HIV-1 infection in Thailand N Engl J Med 2009 361 2209 2220 10.1056/NEJMoa0908492 19843557

[B4] Haynes BF Gilbert PB McElrath MJ Zolla-Pazner S Tomaras GD Alam SM Evans DT Montefiori DC Karnasuta C Sutthent R Immune-correlates analysis of an HIV-1 vaccine efficacy trial N Engl J Med 2012 366 1275 1286 10.1056/NEJMoa1113425 22475592 PMC3371689

[B5] Montefiori DC Karnasuta C Huang Y Ahmed H Gilbert P de Souza MS McLinden R Tovanabutra S Laurence-Chenine A Sanders-Buell E Magnitude and breadth of the neutralizing antibody response in the RV144 and Vax003 HIV-1 vaccine efficacy trials J Infect Dis 2012 Epub ahead of print 10.1093/infdis/jis367 PMC3392187 22634875

[B6] Garcia F Bernaldo de Quiros JC Gomez CE Perdiguero B Najera JL Jimenez V Garcia-Arriaza J Guardo AC Perez I Diaz-Brito V Safety and immunogenicity of a modified pox vector-based HIV/AIDS vaccine candidate expressing Env, Gag, Pol and Nef proteins of HIV-1 subtype B (MVA-B) in healthy HIV-1-uninfected volunteers: a phase I clinical trial (RISVAC02) Vaccine 2011 29 8309 8316 10.1016/j.vaccine.2011.08.098 21907749

[B7] Gomez CE Najera JL Perdiguero B Garcia-Arriaza J Sorzano CO Jimenez V Gonzalez-Sanz R Jimenez JL Munoz-Fernandez MA Lopez Bernaldo de Quiros JC The HIV/AIDS vaccine candidate MVA-B administered as a single immunogen in humans triggers robust, polyfunctional, and selective effector memory T cell responses to HIV-1 antigens J Virol 2011 85 11468 11478 10.1128/JVI.05165-11 21865377 PMC3194965

[B8] Sandstrom E Nilsson C Hejdeman B Brave A Bratt G Robb M Cox J Vancott T Marovich M Stout R Broad immunogenicity of a multigene, multiclade HIV-1 DNA vaccine boosted with heterologous HIV-1 recombinant modified vaccinia virus Ankara J Infect Dis 2008 198 1482 1490 10.1086/592507 18808335 PMC4793972

[B9] Bakari M Aboud S Nilsson C Francis J Buma D Moshiro C Aris EA Lyamuya EF Janabi M Godoy-Ramirez K Broad and potent immune responses to a low dose intradermal HIV-1 DNA boosted with HIV-1 recombinant MVA among healthy adults in Tanzania Vaccine 2011 29 8417 8428 10.1016/j.vaccine.2011.08.001 21864626 PMC4795940

[B10] Thongcharoen P Suriyanon V Paris RM Khamboonruang C de Souza MS Ratto-Kim S Karnasuta C Polonis VR Baglyos L Habib RE A phase 1/2 comparative vaccine trial of the safety and immunogenicity of a CRF01_AE (subtype E) candidate vaccine: ALVAC-HIV (vCP1521) prime with oligomeric gp160 (92TH023/LAI-DID) or bivalent gp120 (CM235/SF2) boost J Acquir Immune Defic Syndr 2007 46 48 55 10.1097/QAI.0b013e3181576795 17909315

[B11] Letourneau S Im EJ Mashishi T Brereton C Bridgeman A Yang H Dorrell L Dong T Korber B McMichael AJ Hanke T Design and pre-clinical evaluation of a universal HIV-1 vaccine PLoS One 2007 2 e984 10.1371/journal.pone.0000984 17912361 PMC1991584

[B12] Rosario M Bridgeman A Quakkelaar ED Quigley MF Hill BJ Knudsen ML Ammendola V Ljungberg K Borthwick N Im EJ Long peptides induce polyfunctional T cells against conserved regions of HIV-1 with superior breadth to single-gene vaccines in macaques Eur J Immunol 2010 40 1973 1984 10.1002/eji.201040344 20468055

[B13] Rossio JL Esser MT Suryanarayana K Schneider DK Bess JW Vasquez GM Wiltrout TA Chertova E Grimes MK Sattentau Q Inactivation of human immunodeficiency virus type 1 infectivity with preservation of conformational and functional integrity of virion surface proteins J Virol 1998 72 7992 8001 9733838 10.1128/jvi.72.10.7992-8001.1998 PMC110135

[B14] Buonaguro L Tagliamonte M Visciano ML Andersen H Lewis M Pal R Tornesello ML Schroeder U Hinkula J Wahren B Buonaguro FM Immunogenicity of HIV virus-like particles in Rhesus Macaques by intra-nasal administration Clin Vaccine Immunol 2012 Epub ahead of print 10.1128/CVI.00068-12 PMC3370444 22461530

[B15] Wang Y Whittall T Rahman D Bunnik EM Vaughan R Scholler J Bergmeier LA Montefiori D Singh M Schuitemaker H Lehner T The role of innate APOBEC3G and adaptive aid immune responses in HLA-HIV/SIV immunized SHIV infected Macaques PLoS One 2012 7 e34433 10.1371/journal.pone.0034433 22514633 PMC3326050

[B16] Liu MA Immunologic basis of vaccine vectors Immunity 2010 33 504 515 10.1016/j.immuni.2010.10.004 21029961

[B17] Mascola JR Montefiori DC The role of antibodies in HIV vaccines Annu Rev Immunol 2010 28 413 444 10.1146/annurev-immunol-030409-101256 20192810

[B18] Negri DR Michelini Z Baroncelli S Spada M Vendetti S Bona R Leone P Klotman ME Cara A Nonintegrating lentiviral vector-based vaccine efficiently induces functional and persistent CD8+ T cell responses in mice J Biomed Biotechnol 2010 2010 534501 20508727 10.1155/2010/534501 PMC2873659

[B19] Laguette N Sobhian B Casartelli N Ringeard M Chable-Bessia C Segeral E Yatim A Emiliani S Schwartz O Benkirane M SAMHD1 is the dendritic- and myeloid-cell-specific HIV-1 restriction factor counteracted by Vpx Nature 2011 474 654 657 10.1038/nature10117 21613998 PMC3595993

[B20] Hallengard D Haller BK Maltais AK Gelius E Nihlmark K Wahren B Brave A Comparison of plasmid vaccine immunization schedules using intradermal in vivo electroporation Clin Vaccine Immunol 2011 18 1577 1581 10.1128/CVI.05045-11 21752954 PMC3165233

[B21] Karim QA Kharsany AB Frohlich JA Baxter C Yende N Mansoor LE Mlisana KP Maarschalk S Arulappan N Grobler A Recruitment of high risk women for HIV prevention trials: baseline HIV prevalence and sexual behavior in the CAPRISA 004 tenofovir gel trial Trials 2011 12 67 10.1186/1745-6215-12-67 21385354 PMC3063209

[B22] no author listed Early end for FEM-PrEP HIV prevention trial. AIDS Patient Care STDS 2011 25 383 21612547 10.1089/apc.2011.9874

[B23] Stolte-Leeb N Loddo R Antimisiaris S Schultheiss T Sauermann U Franz M Mourtas S Parsy C Storer R La Colla P Stahl-Hennig C Topical Nonnucleoside Reverse Transcriptase Inhibitor MC 1220 Partially Prevents Vaginal RT-SHIV Infection of Macaques Aids Research and Human Retroviruses 2011 27 933 943 10.1089/aid.2010.0339 21332419

[B24] Wijesundara DK Jackson RJ Ramshaw IA Ranasinghe C Human immunodeficiency virus-1 vaccine design: where do we go now? Immunol Cell Biol 2011 89 367 374 10.1038/icb.2010.118 20956986

[B25] Kaufman DR Liu J Carville A Mansfield KG Havenga MJ Goudsmit J Barouch DH Trafficking of antigen-specific CD8+ T lymphocytes to mucosal surfaces following intramuscular vaccination J Immunol 2008 181 4188 4198 18768876 10.4049/jimmunol.181.6.4188 PMC2580672

[B26] Pal R Venzon D Santra S Kalyanaraman VS Montefiori DC Hocker L Hudacik L Rose N Nacsa J Edghill-Smith Y Systemic immunization with an ALVAC-HIV-1/protein boost vaccine strategy protects rhesus macaques from CD4+ T-cell loss and reduces both systemic and mucosal simian-human immunodeficiency virus SHIVKU2 RNA levels J Virol 2006 80 3732 3742 10.1128/JVI.80.8.3732-3742.2006 16571790 PMC1440474

[B27] Ranasinghe C Ramshaw IA Immunisation route-dependent expression of IL-4/IL-13 can modulate HIV-specific CD8(+) CTL avidity Eur J Immunol 2009 39 1819 1830 10.1002/eji.200838995 19582753

[B28] Fellay J Shianna KV Ge D Colombo S Ledergerber B Weale M Zhang K Gumbs C Castagna A Cossarizza A A whole-genome association study of major determinants for host control of HIV-1 Science 2007 317 944 947 10.1126/science.1143767 17641165 PMC1991296

[B29] van Manen D Kootstra NA Boeser-Nunnink B Handulle MA van't Wout AB Schuitemaker H Association of HLA-C and HCP5 gene regions with the clinical course of HIV-1 infection AIDS 2009 23 19 28 10.1097/QAD.0b013e32831db247 19050382

[B30] Reinhart D Weik R Kunert R Recombinant IgA production: single step affinity purification using camelid ligands and product characterization J Immunol Methods 2012 378 95 101 10.1016/j.jim.2012.02.010 22570865

[B31] Borrow P Shattock RJ Vyakarnam A Innate immunity against HIV: a priority target for HIV prevention research Retrovirology 2010 7 84 10.1186/1742-4690-7-84 20937128 PMC2964587

[B32] Coma G Pena R Blanco J Rosell A Borras FE Este JA Clotet B Ruiz L Parkhouse RM Bofill M Treatment of monocytes with interleukin (IL)-12 plus IL-18 stimulates survival, differentiation and the production of CXC chemokine ligands (CXCL)8, CXCL9 and CXCL10 Clin Exp Immunol 2006 145 535 544 10.1111/j.1365-2249.2006.03145.x 16907924 PMC1809701

[B33] Kajaste-Rudnitski A Marelli SS Pultrone C Pertel T Uchil PD Mechti N Mothes W Poli G Luban J Vicenzi E TRIM22 inhibits HIV-1 transcription independently of its E3 ubiquitin ligase activity, Tat, and NF-kappaB-responsive long terminal repeat elements J Virol 2011 85 5183 5196 10.1128/JVI.02302-10 21345949 PMC3126207

[B34] Shattock RJ Warren M McCormack S Hankins CA AIDS. Turning the tide against HIV Science 2011 333 42 43 10.1126/science.1206399 21719662

